# Supplementary material for: Saturation time of exposure interval for cross-neutralization response to SARS-CoV-2: Implications for vaccine dose interval
Source: iScience. 2023 Apr 19;26(5):106694. doi: 10.1016/j.isci.2023.106694 (PMC10114312; doi:10.1016/j.isci.2023.106694)
Supplement: Document S1. Figures S1–S3 and Tables S1 and S2 [file mmc1.pdf]

iScience, Volume *26*

Supplemental information

Saturation time of exposure interval for cross-neutralization response to SARS-CoV-2: Implications for vaccine dose interval

Sho Miyamoto, Yudai Kuroda, Takayuki Kanno, Akira Ueno, Nozomi Shiwa-Sudo, Naoko Iwata-Yoshikawa, Yusuke Sakai, Noriyo Nagata, Takeshi Arashiro, Akira Ainai, Saya Moriyama, Noriko Kishida, Shinji Watanabe, Kiyoko Nojima, Yohei Seki, Takuo Mizukami, Hideki Hasegawa, Hideki Ebihara, Shuetsu Fukushi, Yoshimasa Takahashi, Ken Maeda, and Tadaki Suzuki

Supplemental Information
Supplemental Figures

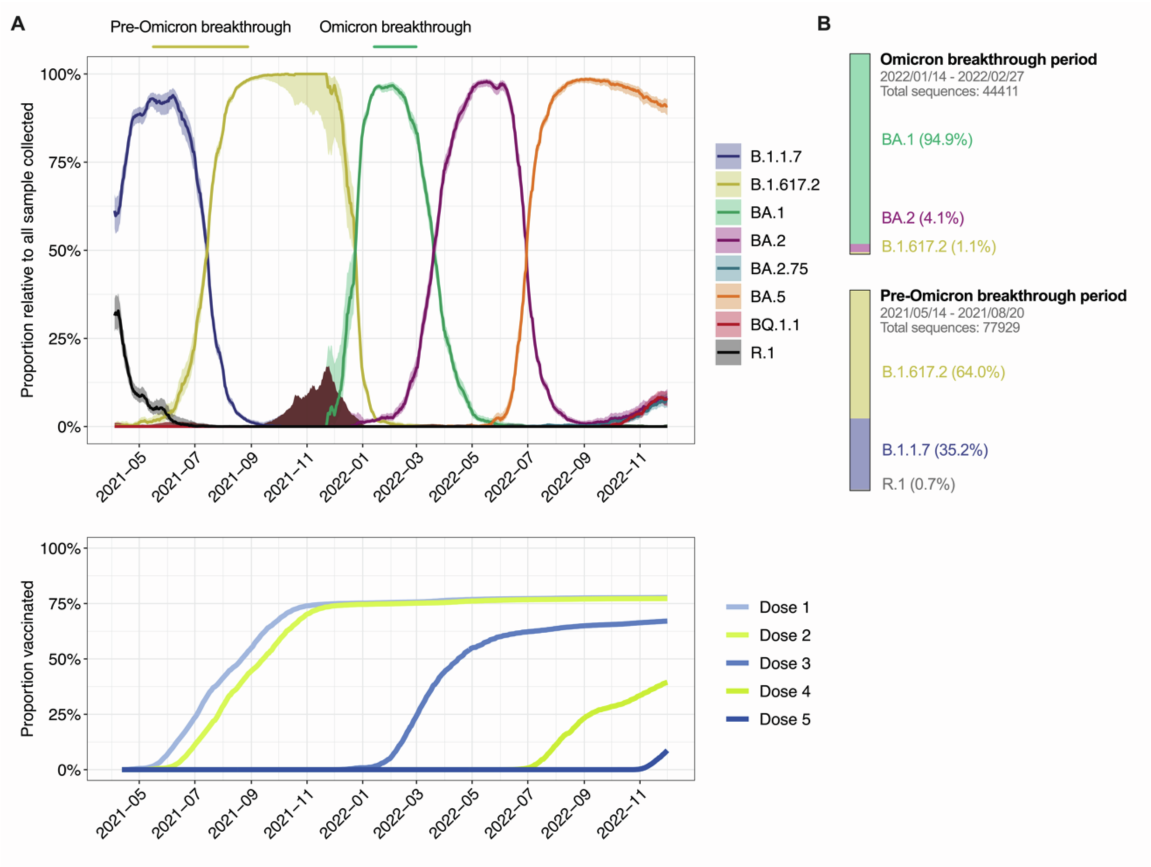

Fig S1. Epigenomic dynamics of SARS-CoV-2 lineages in Japan. Related to Fig 1. (A) The proportion of each variant relative to all sample sequences collected (line) with the 95% confidential interval (ribbon) (upper left), and the vaccination coverage of the population in Japan (lower left) are shown. Among individuals with pre-Omicron and Omicron breakthrough infections, the dates of infection are shown at the top. (B) The proportion of SARS-CoV-2 lineages according to the date of each breakthrough infection. Based on the GISAID database (https://platform.epicov.org), CoV-Spectrum (https://cov-spectrum.org) provided the percentage, the 95% confidence interval, and the number of sequences. Digital Agency, Japan (https://info.vrs.digital.go.jp/dashboard) provided the data on the proportion vaccinated.

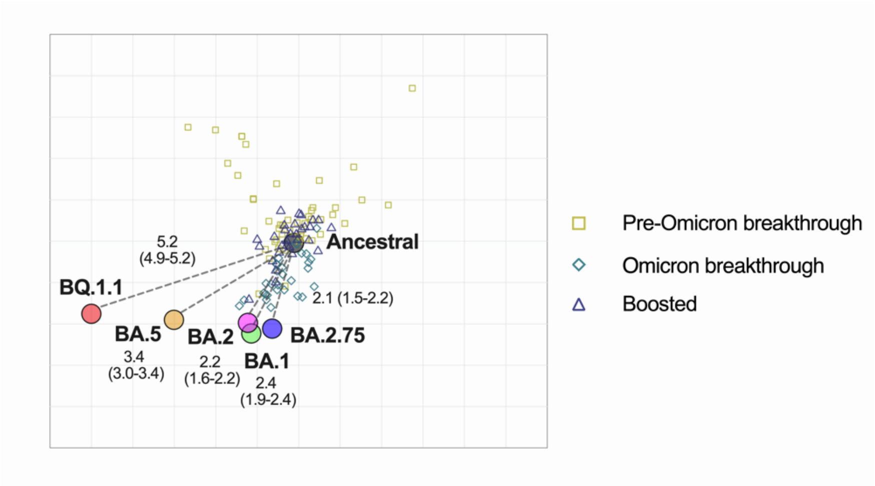

Fig S2. Antigenicity of SARS-CoV-2 Omicron sub-lineages in heterogeneous serum samples from individuals with breakthrough infections and booster vaccine recipients. Related to Fig 1. Antigenic cartography of serum sources for individuals with pre-Omicron/Omicron breakthrough infections and booster vaccine recipients. The variants are shown as circles and serum samples are indicated as squares, diamonds, and triangles. Each square, diamond, and triangle corresponds to a serum sample from one individual. Each grid square (1 antigenic unit) corresponds to a two-fold dilution in the serum sample used in the neutralization assay. Antigenic distance is interpretable in any direction. The median (95% confidence interval) of the distance from the ancestral strain on the map is shown using gray dotted lines.

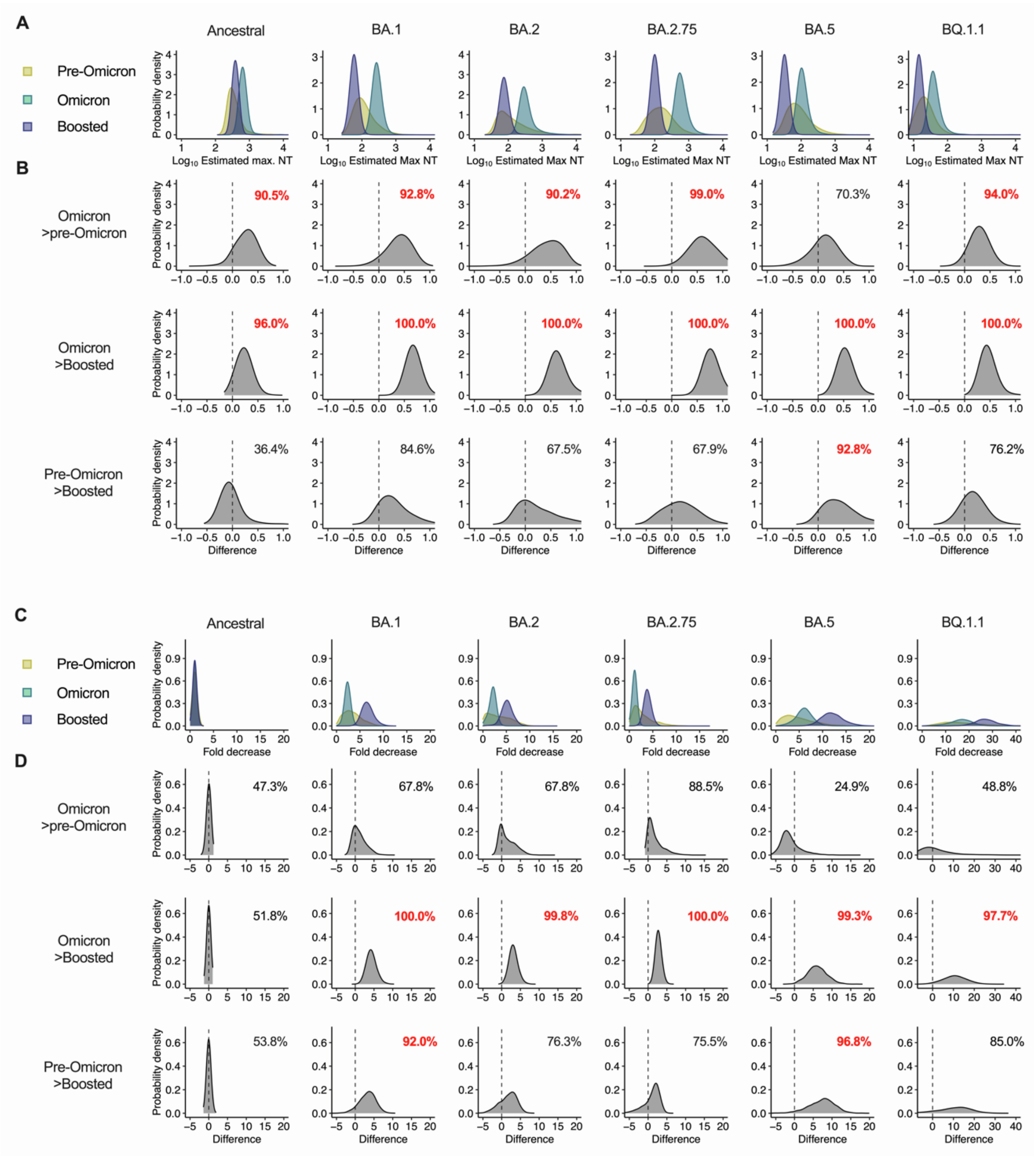

Fig S3. Estimates of the probability that the neutralizing titer and the fold decrease differs according to the exposure history. Related to Fig 3. (A, B) Probability densities of estimated maximum neutralization titers (NTs) against SARS-CoV-2 ancestral strain and Omicron sublineages for each exposure history. (B) Probability density of NT differences between two exposure histories. The indicated probabilities are displayed and highlighted in red if the probability is >90.0%. (C, D) Probability densities of a fold decrease in the NTs relative to the posterior median of NT against the ancestral strain. (D) Probability density of fold decrease differences between two exposure histories. The calculated probabilities are displayed and highlighted in red if the probability is >90.0%.

Supplemental Tables

Table S1. Characteristics of study participants. Related to Fig 1.

	Pre-Omicron breakthrough	Omicron breakthrough	Booster vaccination
**Sample**	Serum	Serum	Serum
**N**	48	30	30
**Age (y)**	36 (26, 50)	48 (38, 54)	44 (37, 52)
**Male sex**	14 (29%)	8 (27%)	4 (13%)
**Vaccine (2 doses)**			
BNT162b2	47 (98%)	19 (63%)	30 (100%)
mRNA-1273	0 (0%)	11 (37%)	0 (0%)
Not listed	1 (2.1%)	0 (0%)	0 (0%)
**Dose 1 to dose 2 interval (days)**	21 (21, 21)	21 (21, 28)	21 (21, 21)
**Dose 2 to infection (days)**	44 (30, 62)	166 (134, 177)	NA
**Dose 2 to dose 3 interval (days)**	NA	NA	237 (223, 254)
**Last exposure to collection (days)**	14 (11, 15)	20 (14, 24)	19 (15, 25)
**Period of infection**	May. 14 - Aug. 20, 2021	Jan. 15 - Feb. 27, 2022	NA

Median (interquartile range); n (%)

Table S2. Means of the saturated neutralization titers against SARS-CoV-2 variants. Related to Fig 2.

Target virus	Exposure-history	Posterior mean	Posterior 2.5%	Posterior 97.5%	R-hat	Effective sample size
Ancestral	Total	2.65	2.21	3.14	1.00	2092
Ancestral	Pre-Omicron breakthrough	2.56	2.26	3.14	1.00	1165
Ancestral	Omicron breakthrough	2.82	2.61	3.06	1.00	1039
Ancestral	Booster vaccination	2.59	2.41	2.77	1.00	1736
BA.1	Total	2.13	1.55	2.82	1.00	3267
BA.1	Pre-Omicron breakthrough	2.06	1.62	2.79	1.00	3320
BA.1	Omicron breakthrough	2.45	2.26	2.74	1.00	2468
BA.1	Booster vaccination	1.77	1.61	1.94	1.00	3368
BA.2	Total	2.21	1.56	3.12	1.00	1954
BA.2	Pre-Omicron breakthrough	2.13	1.58	3.30	1.00	1465
BA.2	Omicron breakthrough	2.54	2.28	3.25	1.00	1291
BA.2	Booster vaccination	1.89	1.70	2.15	1.00	1882
BA.2.75	Total	2.34	1.67	3.07	1.00	3758
BA.2.75	Pre-Omicron breakthrough	2.18	1.59	2.95	1.00	3413
BA.2.75	Omicron breakthrough	2.78	2.55	3.21	1.00	3165
BA.2.75	Booster vaccination	2.00	1.84	2.18	1.00	3527
BA.5	Total	1.89	1.34	2.65	1.00	2978
BA.5	Pre-Omicron breakthrough	1.94	1.41	2.79	1.00	3036
BA.5	Omicron breakthrough	2.05	1.83	2.46	1.00	2475
BA.5	Booster vaccination	1.51	1.35	1.70	1.00	3282
BQ.1.1	Total	1.44	0.95	2.17	1.00	3140
BQ.1.1	Pre-Omicron breakthrough	1.33	0.87	1.92	1.00	2655
BQ.1.1	Omicron breakthrough	1.63	1.41	2.08	1.00	1207
BQ.1.1	Booster vaccination	1.16	1.02	1.35	1.00	1204
